# LncRNA ELF3-AS1 is a Prognostic Biomarker and Correlated with Immune Infiltrates in Hepatocellular Carcinoma

**DOI:** 10.1155/2021/8323487

**Published:** 2021-07-09

**Authors:** Tianming Chen, Changhao Zhu, Xing Wang, Yaozhen Pan

**Affiliations:** ^1^Department of General Surgery, Gulou Hospital, School of Medicine, Nanjing University, Nanjing 210008, Jiangsu, China; ^2^The Affiliated Cancer Hospital of Guizhou Medical University, Guiyang 550001, Guizhou, China; ^3^College of Clinical Medicine, Guizhou Medical University, Guiyang 550000, Guizhou, China

## Abstract

**Background:**

Expression of long noncoding RNA (lncRNA) ELF3 antisense RNA 1 (ELF3-AS1) is observed in some cancers, while its role in hepatocellular carcinoma (HCC) is unclear. The study aimed to investigate the relationship between ELF3-AS1 and HCC based on database, bioinformatics, and statistical analysis.

**Methods:**

In this study, Kruskal–Wallis test, Wilcoxon sign-rank test, logistic regression, Kaplan–Meier method, Cox regression analysis, gene set enrichment analysis (GSEA), and immunoinfiltration analysis were used to assess the relationship between ELF3-AS1 expression and clinical characteristics of HCC patients, the relationship between ELF3-AS1 expression and prognosis of HCC patients, and the possible functions of ELF3-AS1 in HCC.

**Results:**

High expression of ELF3-AS1 in patients with HCC was related to *T* stage (*P* < 0.001), gender (*P* = 0.006), residual tumor (*P* = 0.008), histologic grade (*P* < 0.001), adjacent hepatic tissue inflammation (*P* = 0.011), AFP (*P* < 0.001), and vascular invasion (*P* = 0.028). High ELF3-AS1 expression was associated with poor overall survival (OS) (*P* = 0.001) and DSS (*P* = 0.047). ELF3-AS1 expression (*P* = 0.011) was independently correlated with OS in HCC patients. In the high ELF3-AS1 expression group, GPCR-radioligand binding, *M* phase, Class A/1 (rhodopsin-like receptors), cell cycle checkpoints, translation, mitotic metaphase and anaphase, signaling by robo receptors, keratinization, and rRNA processing were differentially enriched by GESA. ELF3-AS1 expression was associated with immune infiltrating cells.

**Conclusions:**

ELF3-AS1 expression was associated with poor prognosis in HCC. ELF3-AS1 expression was significantly associated with immune infiltration. ELF3-AS1 is a promising biomarker that can be used for the diagnosis and prognosis of HCC.

## 1. Introduction

Hepatocellular carcinoma (HCC) is one of the leading causes of cancer deaths in the world [1]. In the last few years, more than 700,000 people have died each year from HCC, and this number is increasing every year [2]. HCC is a complex neoplastic disease that is highly aggressive, with a pathology that includes changes in tumor cell behavior and the vascular system [3]. Nowadays, surgery is only effective for patients with early HCC [4]. Most patients are diagnosed at an advanced stage, and radiotherapy and chemotherapy are not effective [4]. The high recurrence and metastasis rates of HCC reduce patient survival [5]. Despite some advances in surgical treatment and neoadjuvant therapy, the prognosis of HCC is poor [6]. The lack of useful markers makes it difficult for clinicians to predict the clinical outcome of patients with HCC. Therefore, new markers need to be explored to identify HCC patients with poor prognosis.

The length of long noncoding RNAs (lncRNAs) is over 200 bp [7]. lncRNAs play a key role in the formation of cancer [8]. Growing evidence for the involvement of aberrant lncRNAs in the development and progression of HCC [4, 6, 9, 10]. Therefore, screening for lncRNAs that are clinically relevant to HCC is important for the early diagnosis and effective treatment of HCC.

The lncRNA ELF3 antisense RNA 1 (ELF3-AS1) is the antisense transcript for ELF3. ELF3-AS1 was used as an oncogene in bladder cancer (BLCA) [11]. ELF3-AS1 is upregulated in oral squamous cell carcinoma (OSCC) [12]. ELF3-AS1 is upregulated in osteosarcoma (OS) [13]. ELF3-AS1 is upregulated in nonsmall cell lung cancer (NSCLC) [14]. ELF3-AS1 is a prognostic marker and potential therapeutic target for gliomas [15]. However, the correlation between ELF3-AS1 and HCC has not been studied.

In this study, we compared the differences in ELF3-AS1 expression between HCC tumor tissues and normal samples, explored the correlation between ELF3-AS1 expression and clinical characteristics of HCC, and assessed the prognostic value of ELF3-AS1 in HCC. For ELF3-AS1 high and low expression groups, genomic enrichment analysis (GSEA) was performed to explore the possible functions of ELF3-AS1. The possible function of ELF3-AS1 in HCC was explored by immune infiltration analysis. The study may provide new directions for the development of diagnostic and therapeutic strategies for HCC.

## 2. Materials and Methods

### 2.1. Differential Expression of ELF3-AS1

#### 2.1.1. Baseline Information

Software used was R (version 3.6.3) (statistical analysis and visualization). R package type was basic R package. The molecule is ELF3-AS1 (ENSG00000234678). The subgroup is median. Data are RNAseq data and clinical data in level 3 HTSeq-FPKM format from the TCGA (https://portal.gdc.cancer.gov) HCC (hepatocellular carcinoma) project. Data filtering is through retaining data with clinical information. Data conversion included RNAseq data in FPKM (Fragments Per Kilobase per Million) format converted to TPM (transcripts per million reads) format and grouped according to molecular expression.

#### 2.1.2. Unpaired Samples

Software used was R (version 3.6.3). R package type was mainly ggplot2. Molecules used were ELF3-AS1. Data are RNAseq data in level 3 HTSeq-FPKM format from the TCGA HCC project. Data filtering is none. Data transformation included FPKM format RNAseq data converted to TPM format and log2 transformed for sample-to-sample expression comparisons.

#### 2.1.3. Paired Samples

Software used was R (version 3.6.3). R package was mainly ggplot2. Molecules included ELF3-AS1. Data are RNAseq data in level 3 HTSeq-FPKM format from the TCGA HCC project. Data filtering is through retaining paired samples. Data transformation included RNAseq data in FPKM format converted to TPM format and log2 transformed for sample-to-sample expression comparisons.

#### 2.1.4. ROC Analysis

Software used was R (version 3.6.3). R packages used were pROC package (for analysis) || ggplot2 package. Molecule is ELF3-AS1. Clinical variables are tumor vs. normal. Data are RNAseq data and clinical data in level 3 HTSeq-FPKM format from the TCGA HCC project. Data filtering is through retaining data with clinical information. Data transformation included RNAseq data in FPKM format converted to TPM format and log2 transformed for analysis. ROC results were interpreted as false positive rate (FPR) is the horizontal coordinate and true positive rate (TPR) is the vertical coordinate.

### 2.2. The Relationship between ELF3-AS1 and Clinical Characteristics

#### 2.2.1. Correlation of Gene Expression with Clinical Characteristics

Software used was R (version 3.6.3). R package mainly included ggplot2. Molecule is ELF3-AS1. Clinical variables: *T* stage, gender, residual tumor, histological grade, adjacent hepatic tissue inflammation, AFP, and vascular invasion. Data are RNAseq data and clinical data in level 3 HTSeq-FPKM format from the TCGA HCC project. Data filtering is through retaining data with clinical information. Data transformation included RNAseq data in FPKM format converted to TPM format and log2 transformed for analysis.

#### 2.2.2. Logistics Analysis

Software used was R (version 3.6.3). R package type was mainly the basic package. Statistical method is the dichotomous logistic model. Dependent variable is ELF3-AS1. Type of independent variable is low high dichotomous. Data are RNAseq data in level 3 HTSeq-FPKM format from the TCGA HCC project. Data filtering is through retaining data with clinical information. Data conversion included RNAseq data in FPKM format converted to TPM format and grouped according to molecular expression.

### 2.3. The Relationship between ELF3-AS1 and Prognosis

#### 2.3.1. Kaplan–Meier Method

Software used was R (version 3.6.3). R packages are survminer package and survival package. Molecule is ELF3-AS1. Subgroups are 0–50 vs. 50–100. Prognosis type is overall survival and disease specific survival. Data are RNAseq data and clinical data in level 3 HTSeq-FPKM format from the TCGA HCC project. Data filtering is through retaining data with clinical information. Data conversion included RNAseq data in FPKM format converted to TPM format and analyzed by grouping them according to molecular expression. Additional data are prognostic data from an article [16].

#### 2.3.2. COX Regression

Software used was R (version 3.6.3). R package is survivor package. Statistical method is COX regression module. Prognosis type is overall survival. Included variables are clinical characteristics and ELF3-AS1. Data are RNAseq data and clinical data in level 3 HTSeq-FPKM format from the TCGA HCC project. Data filtering is through retaining data with clinical information. Data conversion included RNAseq data in FPKM format converted to TPM format and analyzed by grouping them according to molecular expression. Additional data are prognostic data from an article [16].

### 2.4. Enrichment of ELF3-AS1-Related Pathways

#### 2.4.1. Single Gene Differential Analysis

Software used was R (version 3.6.3). R package is deseq2 [17]. Target molecule is ELF3-AS1. Low expression group is 0–50%. High expression group is 50–100%. Data are RNAseq data in level 3 HTSeq-Counts format from the TCGA HCC project. Data filtering is filtering of paracancer samples.

#### 2.4.2. GSEA Analysis

Software used was R (version 3.6.3). R package are ggplot2 package and clusterProfiler package [18] (for GSEA analysis). Method is gene set enrichment analysis (GSEA) [19]. Species is *Homo sapiens*. Reference gene collection is c2.cp.v7.2.symbols.gmt (curated). Gene set database is MSigDB Collections (database hyperlink) (with detailed descriptions of individual gene sets).

### 2.5. The Relationship between ELF3-AS1 Expression and Immune Infiltrating Cells

Software used was R (version 3.6.3). R package is GSVA package [20]. Immunocell algorithm is ssGSEA (algorithm built into the GSVA package). Molecule is ELF3-AS1. Immune cells are 24 immune cells. Data are RNAseq data and clinical data in level 3 HTSeq-FPKM format from the TCGA HCC project. Data filtering is removal of paracancerous tissue. Expression profile data conversion included FPKM format RNAseq data converted to TPM format and log2 transformed for analysis. Other data included markers for 24 immune cells obtained from an article [21].

## 3. Results

### 3.1. Clinical Characteristics

As shown in Table 1, the characteristics of HCC in TCGA were collected. The age included 177 patients (≤60, 47.5%) and 196 patients (>60, 52.5%). The median age is 61 years, with a range of 52 to 69 years. The race included 185 white patients, 160 Asian patients, and 17 Black or African American patients. The pathologic stage included 173 stage I (49.4%), 87 stage II (24.9%), 85 stage III (24.3%), and 5 stage IV (1.4%). The tumor status included 202 tumor-free (56.9%) and 153 with tumor (43.1%). The gender included 121 females (32.4%) and 253 males (67.6%). The weight included 184 (≤70) (53.2%) and 162 (>70) (46.8%). The height included 201 (<170) (58.9%) and 140 (≥170) (41.1%). The BMI included 177 (≤25) (52.5%) and 160 (>25) (47.5%). The residual tumor included 327 R0 (94.8%), 17 R1 (4.9%), and 1 R2 (0.3%). The histologic grade included 55 G1 (14.9%), 178 G2 (48.2%), 124 G3 (33.6%), and 12 G4 (3.3%). The adjacent hepatic tissue inflammation included 118 none (49.8%), 101 mild (42.6%), and 18 severe (7.6%). The albumin included 69 (<3.5) (23%) and 231 (≥3.5) (77%). The AFP included 215 (≤400) (76.8%) and 65 (>400) (23.2%). The prothrombin time included 208 (≤4) (70%) and 89 (>4) (30%). The Child-Pugh grade included 219 A (90.9%), 21 B (8.7%), and 1 C (0.4%). The fibrosis Ishak score included 75 0 (34.9%), 31 1/2 (14.4%), 28 3/4 (13%), and 81 5/6 (37.7%). The vascular invasion included 208 no (65.4%) and 110 yes (34.6%).

### 3.2. ELF3-AS1 Expression Correlated with Poor Clinical Characteristics of HCC

**Figure 1:**
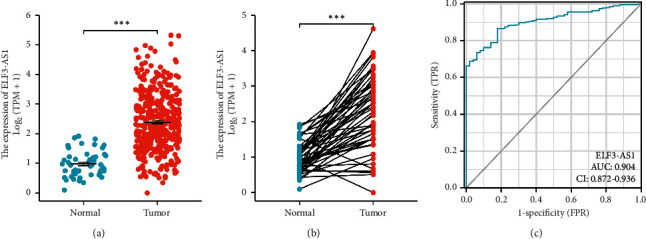
Expression of ELF3-AS1 in HCC and normal or matched normal liver tissues. (a) HCC tissues and normal liver tissues. (b) HCC tissues and matched normal liver tissues. (c) ROC curve.

**Figure 2:**
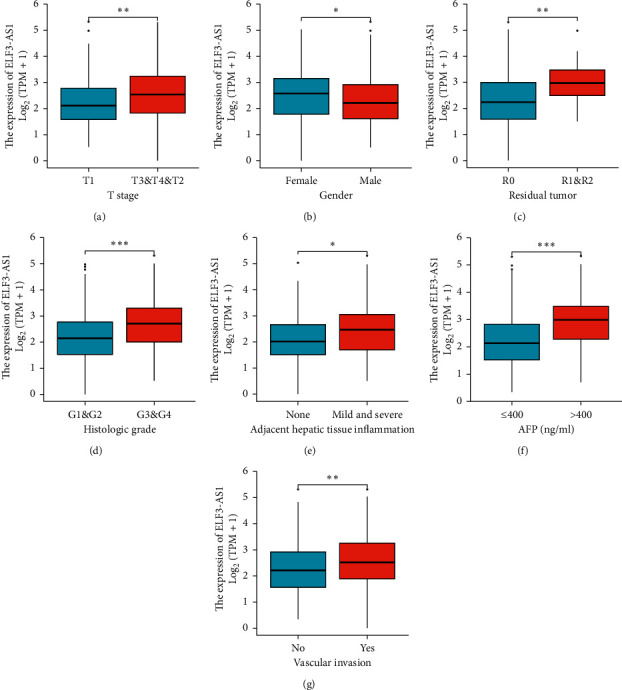
ELF3-AS1 expression in HCC was significantly associated with clinical characteristics. (a) T stage. (b) Gender. (c) Residual tumor. (d) Histological grade. (e) Adjacent hepatic tissue inflammation. (f) AFP. (g) Vascular invasion.

As shown in Figure 1(a), ELF3-AS1 was highly expressed in HCC tissues (0.978 ± 0.061 vs. 2.381 ± 0.052, *P* < 0.001). As shown in Figure 1(b), LF3-AS1 was highly expressed in HCC tissues (*P* < 0.001), based on 50 HCC tissues and their matched normal liver tissues. As shown in Figure 1(c), the AUC of ELF3-AS1 was 0.904. As shown in Table 2, clinical and gene expression data were collected. As shown in Table 2, the *P* value of *T* stage is 0.002, the *P* value of pathologic stage is 0.003, the *P* value of gender is 0.008, the *P* value of race is 0.037, the *P* value of residual tumor is *P* = 0.004, the *P* value of histologic grade is <0.001, the *P* value of adjacent hepatic tissue inflammation is 0.025, the *P* value of AFP is <0.001, and the *P* value of vascular invasion is 0.037. As shown in Figure 2 and Table 3, the *P* value of *T* stage is <0.001, the *P* value of gender is 0.006, the *P* value of residual tumor is 0.008, the *P* value of histologic grade is <0.001, the *P* value of adjacent hepatic tissue inflammation is 0.011, the *P* value of AFP is <0.001, and the *P* value of vascular invasion is 0.028.

### 3.3. Relationship between ELF3-AS1 and Survival of HCC Patients

**Figure 3:**
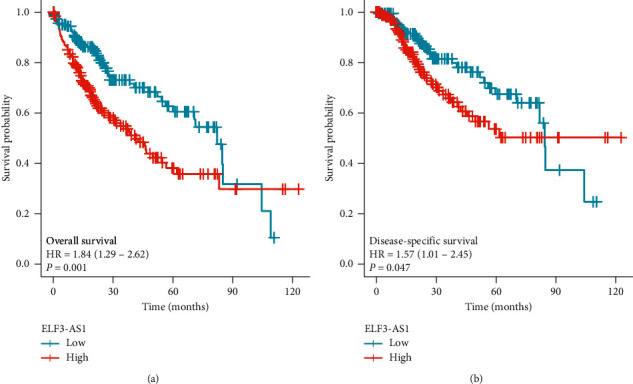
Low ELF3-AS1 expression was associated with poor OS and DSS in patients with HCC. (a) Over survival. (b) Disease Specific Survival.

As shown in Figure 3, the expression of ELF3-AS1 was positively correlated with poor OS (*P* = 0.001), disease-specific survival (DSS) (*P* = 0.047) of HCC patients. As shown in Table 4, the results of univariate analysis showed that high ELF3-AS1 expression levels (*P* < 0.001) were associated with *T* stage (*P* < 0.001), pathologic stage (*P* < 0.001), and tumor status (*P* < 0.001). The results of multivariate analysis showed that ELF3-AS1 expression (*P* = 0.011), pathologic stage (*P* = 0.013), age (*P* = 0.013), and tumor status (*P* = 0.005) were independently correlated with OS in multivariate analysis. The results suggested that increased expression of ELF3-AS1 was associated with poor OS.

### 3.4. ELF3-AS1-Related Pathways

**Figure 4:**
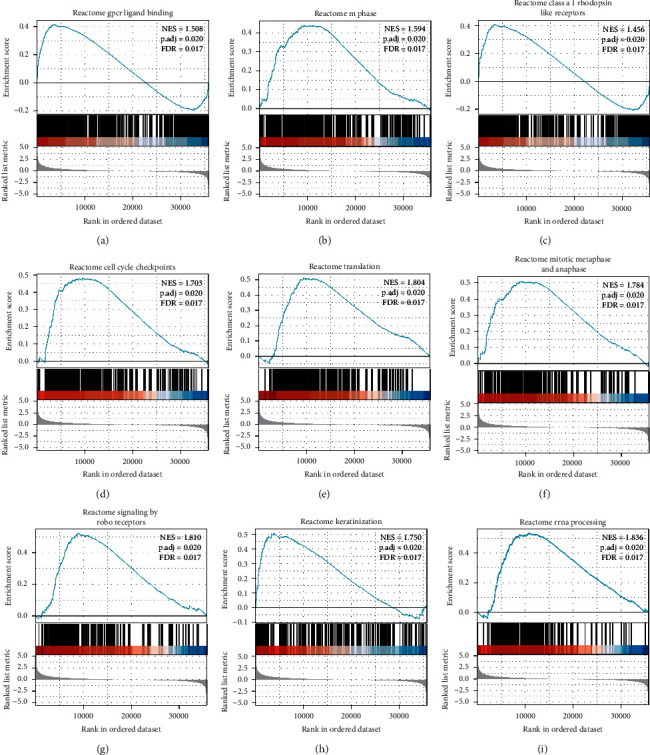
GSEA of ELF3-AS1 in HCC. (a) GPCR-radioligand binding, (b) M phase, (c) Class A/1 (rhodopsin-like receptors), (d) cell cycle checkpoints, (e) translation, (f) mitotic metaphase and anaphase, (g) signaling by robo receptors, (h) keratinization, and (i) rRNA processing were enriched in ELF3-AS1-related HCC. NES, normalized ES; FDR, false discovery rate.

A dataset of 191 significant differences was enriched in ELF3-AS1 low expression phenotypes. As shown in Table 5 and Figure 4, the top 9 low *P*-value datasets include GPCR-radioligand binding, *M* phase, Class A/1 (rhodopsin-like receptors), cell cycle checkpoints, translation, mitotic metaphase and anaphase, signaling by robo receptors, keratinization, and rRNA processing.

### 3.5. Correlation of ELF3-AS1 Expression with Immune Infiltration

**Figure 5:**
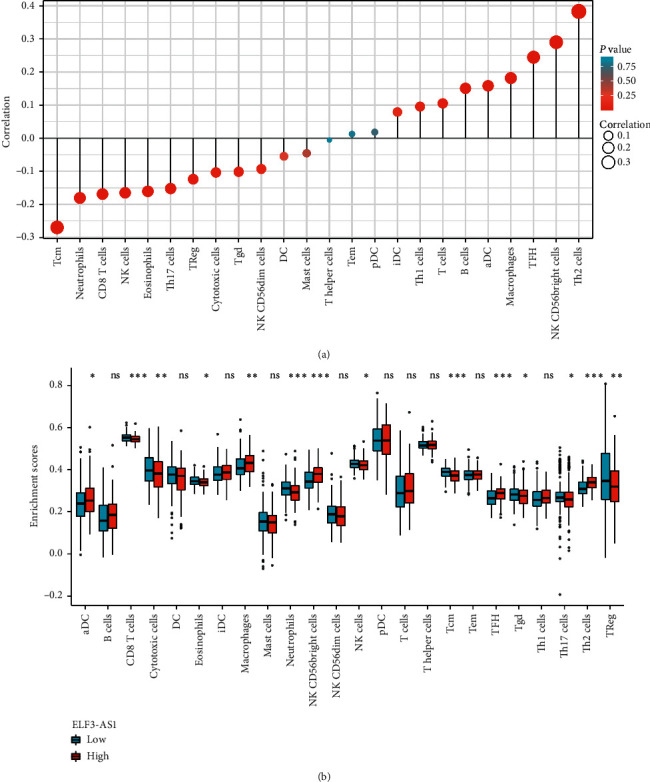
ELF3-AS1 expression is associated with immune infiltration in the tumor microenvironment of HCC. (a) Correlation between ELF3-AS1 expression and 24 immune cells (Spear). (b) Differences in immune cell enrichment scores between ELF3-AS1 high and low expression groups (Wilcoxon rank sum test).

As shown in Figure 5 and Table 6, ELF3-AS1 expression is negatively and significantly correlated with infiltration levels of CD8 T cells (*P* = 0.001), cytotoxic cells (*P* = 0.045), eosinophils (*P* = 0.002), neutrophils (*P* < 0.001), NK cells (*P* = 0.001), Tcm (*P* < 0.001), Th17 cells (*P* = 0.003), and TReg (*P* = 0.017) and positively correlated with that of aDC (*P* = 0.002), B cells (*P* = 0.004), macrophages (*P* < 0.001), NK CD56bright cells (*P* < 0.001), T cells (*P* = 0.042), TFH (*P* < 0.001), and Th2 cells (*P* < 0.001).

## 4. Discussion

LncRNAs play a key role in tumorigenesis and progression [22]. ZNF385D-AS2 may be a useful biomarker for prognosis in patients with HCC [9]. High LUCAT1 expression is an independent prognostic factor for HCC [6]. F11-AS1 may serve as a therapeutic target for HCC [4]. GIHCG is a biomarker that can be used to predict the prognosis of patients with HCC [10]. Therefore, the study of lncRNAs as new HCC biomarkers and therapeutic targets is very important.

ELF3-AS1 is upregulated in OSCC, OS, and NSCLC [12–14]. In this study, high ELF3-AS1 expression in HCC was associated with the characteristics including *T* stage, gender, residual tumor, histologic grade, adjacent hepatic tissue inflammation, AFP, and vascular invasion. High ELF3-AS1 expression was associated with poor OS and DSS. ELF3-AS1 expression was independently correlated with OS in HCC patients.

ELF3-AS1 mediated BLCA tumorigenesis through enhanced ELF3-AS1/KLF8 signaling [11]. ELF3-AS1 may be regulating GLUT1 to promote the proliferation of OSCC cells [12]. ELF3-AS1 may promote OS cell proliferation by upregulating KLF12 through methylation of the miR-205 gene [13]. ELF3-AS1 promotes cancer cell invasion and migration through downregulation of miR-212 by methylation in NSCLC [14]. ELF3-AS1 is not only an important prognostic marker, but also a potential therapeutic target for glioma [15]. In this study, based on GESA, ELF3-AS1 was related to pathways including GPCR-radioligand binding, *M* phase, Class A/1 (rhodopsin-like receptors), cell cycle checkpoints, translation, mitotic metaphase and anaphase, signaling by robo receptors, keratinization, and rRNA processing.

Immune infiltrating cells in HCC are currently a hot topic, and a major understanding of immune infiltrating cells can support the development of new and emerging immunotherapies. The development and progression of HCC are associated with a unique immune response profile of the liver microenvironment, in which CD4+CD25+Foxp3 regulatory T cells (Tregs) play a key role through their immunosuppressive effects [23]. Immunotherapy enhances the immune response against hepatocellular carcinoma by blocking Treg activity [23]. In this study, we explored the relationship between ELF3-AS1 expression in HCC and various immune infiltrations. ELF3-AS1 expression was associated with infiltration of CD8 T cells, cytotoxic cells, eosinophils, neutrophils, NK cells, Tcm, Th17 cells, Treg, aDC, B cells, macrophages, NK CD56bright cells, T cells, TFH, and Th2 cells in HCC. These correlations may suggest potential mechanisms by which ST-AS1 inhibits the function of CD8 T cells, cytotoxic cells, eosinophils, neutrophils, NK cells, Tcm, Th17 cells, and Treg and subsequently promotes the function of aDC, B cells, macrophages, NK CD56bright cells, T cells, TFH, and Th2 cells.

Despite some limitations, this is an exploratory study to investigate the relationship between ELF3-AS1 and HCC. This study is based on database and bioinformatics analysis. We have not yet investigated in detail the direct mechanism of ELF3-AS1 involvement in HCC. Further studies are needed regarding the direct mechanism of ELF3-AS1-mediated HCC.

## 5. Conclusion

ELF3-AS1 was highly expressed in HCC relative to normal tissue and related to poor OS and DSS. ELF3-AS1 might participate in the development of HCC by pathways including GPCR-radioligand binding, *M* phase, Class A/1 (rhodopsin-like receptors), cell cycle checkpoints, translation, mitotic metaphase and anaphase, signaling by robo receptors, keratinization, and rRNA processing. ELF3-AS1 expression was associated with immune infiltrating cells. This study investigated the role of ELF3-AS1 in HCC and provided a promising biomarker for the diagnosis and prognosis of HCC.

## Figures and Tables

**Table 1:** Characteristics of patients with HCC (TCGA).

Characteristic	Levels	Overall
*n*		374
*T* stage, *n* (%)	*T*1	183 (49.3%)
	*T*2	95 (25.6%)
	*T*3	80 (21.6%)
	*T*4	13 (3.5%)

*N* stage, *n* (%)	*N*0	254 (98.4%)
	*N*1	4 (1.6%)

*M* stage, *n* (%)	*M*0	268 (98.5%)
	*M*1	4 (1.5%)

Pathologic stage, *n* (%)	Stage I	173 (49.4%)
	Stage II	87 (24.9%)
	Stage III	85 (24.3%)
	Stage IV	5 (1.4%)

Tumor status, *n* (%)	Tumor free	202 (56.9%)
	With tumor	153 (43.1%)

Gender, *n* (%)	Female	121 (32.4%)
	Male	253 (67.6%)

Race, *n* (%)	Asian	160 (44.2%)
	Black or African American	17 (4.7%)
	White	185 (51.1%)

Age, *n* (%)	≤60	177 (47.5%)
	>60	196 (52.5%)

Weight, *n* (%)	≤70	184 (53.2%)
	>70	162 (46.8%)

Height, *n* (%)	<170	201 (58.9%)
	≥170	140 (41.1%)

BMI, *n* (%)	≤25	177 (52.5%)
	>25	160 (47.5%)

Residual tumor, *n* (%)	R0	327 (94.8%)
	R1	17 (4.9%)
	R2	1 (0.3%)

Histologic grade, *n* (%)	G1	55 (14.9%)
	G2	178 (48.2%)
	G3	124 (33.6%)
	G4	12 (3.3%)

Adjacent hepatic tissue inflammation, *n* (%)	None	118 (49.8%)
	Mild	101 (42.6%)
	Severe	18 (7.6%)

Albumin (g/dl), *n* (%)	<3.5	69 (23%)
	≥3.5	231 (77%)

AFP (ng/ml), *n* (%)	≤400	215 (76.8%)
	>400	65 (23.2%)

Prothrombin time, *n* (%)	≤4	208 (70%)
	>4	89 (30%)

Child-Pugh grade, *n* (%)	*A*	219 (90.9%)
	*B*	21 (8.7%)
	*C*	1 (0.4%)

Fibrosis Ishak score, *n* (%)	0	75 (34.9%)
	1/2	31 (14.4%)
	3/4	28 (13%)
	5/6	81 (37.7%)

Vascular invasion, *n* (%)	No	208 (65.4%)
	Yes	110 (34.6%)

Age, median (IQR)		61 (52, 69)

**Table 2:** Correlation between ELF3-AS1 expression and clinical characteristics of HCC.

Characteristic	Low expression of ELF3-AS1	High expression of ELF3-AS1	*P*
n	187	187	
*T* stage, *n* (%)			**0.002**
*T*1	107 (28.8%)	76 (20.5%)	
*T*2	36 (9.7%)	59 (15.9%)	
*T*3	38 (10.2%)	42 (11.3%)	
*T*4	3 (0.8%)	10 (2.7%)	

*N* stage, *n* (%)			0.623
*N*0	124 (48.1%)	130 (50.4%)	
*N*1	1 (0.4%)	3 (1.2%)	

*M* stage, *n* (%)			0.124
*M*0	129 (47.4%)	139 (51.1%)	
*M*1	0 (0%)	4 (1.5%)	

Pathologic stage, *n* (%)			**0.003**
Stage I	102 (29.1%)	71 (20.3%)	
Stage II	33 (9.4%)	54 (15.4%)	
Stage III	38 (10.9%)	47 (13.4%)	
Stage IV	1 (0.3%)	4 (1.1%)	

Tumor status, *n* (%)			0.101
Tumor free	110 (31%)	92 (25.9%)	
With tumor	69 (19.4%)	84 (23.7%)	

Gender, *n* (%)			**0.008**
Female	48 (12.8%)	73 (19.5%)	
Male	139 (37.2%)	114 (30.5%)	

Race, *n* (%)			**0.037**
Asian	70 (19.3%)	90 (24.9%)	
Black or African American	12 (3.3%)	5 (1.4%)	
White	100 (27.6%)	85 (23.5%)	

Age, *n* (%)			0.277
≤60	83 (22.3%)	94 (25.2%)	
>60	104 (27.9%)	92 (24.7%)	

Weight, *n* (%)			0.272
≤70	88 (25.4%)	96 (27.7%)	
>70	88 (25.4%)	74 (21.4%)	

Height, *n* (%)			0.130
<170	94 (27.6%)	107 (31.4%)	
≥170	78 (22.9%)	62 (18.2%)	
BMI, *n* (%)			0.296
≤25	84 (24.9%)	93 (27.6%)	
>25	86 (25.5%)	74 (22%)	

Residual tumor, *n* (%)			**0.004**
*R*0	172 (49.9%)	155 (44.9%)	
*R*1	3 (0.9%)	14 (4.1%)	
*R*2	0 (0%)	1 (0.3%)	

Histologic grade, *n* (%)			**< 0.001**
*G*1	41 (11.1%)	14 (3.8%)	
*G*2	94 (25.5%)	84 (22.8%)	
*G*3	47 (12.7%)	77 (20.9%)	
*G*4	2 (0.5%)	10 (2.7%)	

Adjacent hepatic tissue inflammation, *n* (%)			**0.025**
None	73 (30.8%)	45 (19%)	
Mild	44 (18.6%)	57 (24.1%)	
Severe	10 (4.2%)	8 (3.4%)	

Albumin(g/dl), *n* (%)			0.982
<3.5	36 (12%)	33 (11%)	
≥3.5	118 (39.3%)	113 (37.7%)	

AFP (ng/ml), *n* (%)			**< 0.001**
≤400	119 (42.5%)	96 (34.3%)	
>400	18 (6.4%)	47 (16.8%)	

Prothrombin time, *n* (%)			0.889
≤4	104 (35%)	104 (35%)	
>4	46 (15.5%)	43 (14.5%)	

Child-Pugh grade, *n* (%)			0.176
*A*	110 (45.6%)	109 (45.2%)	
*B*	14 (5.8%)	7 (2.9%)	
*C*	1 (0.4%)	0 (0%)	

Fibrosis Ishak score, *n* (%)			0.526
0	43 (20%)	32 (14.9%)	
1/2	13 (6%)	18 (8.4%)	
3/4	15 (7%)	13 (6%)	
5/6	45 (20.9%)	36 (16.7%)	

Vascular invasion, *n* (%)			**0.037**
No	114 (35.8%)	94 (29.6%)	
Yes	46 (14.5%)	64 (20.1%)	

Age, median (IQR)	63 (53, 69)	60 (51, 68)	0.239

**Table 3:** ELF3-AS1 expression was associated with clinical characteristics of HCC (logistic regression).

Characteristics	Total (*N*)	Odds ratio (OR)	*P* value
*T* stage (*T*2 & *T*3 & *T*4 vs. *T*1)	371	2.030 (1.345–3.078)	**<0.001**
*N* stage (*N*1 vs. *N*0)	258	2.862 (0.361–58.271)	0.365
*M* stage (*M*1 vs. *M*0)	272	66845126.402 (0.000-NA)	0.994
Pathologic stage (Stage III & Stage IV vs. Stage I & Stage II)	350	1.412 (0.873–2.298)	0.161
Tumor status (with tumor vs. tumor free)	355	1.456 (0.955–2.224)	0.081
Gender (male vs. female)	374	0.539 (0.346–0.835)	**0.006**
Race (Asian and black or African American vs. white)	362	1.363 (0.902–2.064)	0.142
Age (>60 vs. ≤60)	373	0.781 (0.519–1.173)	0.234
Weight (>70 vs. ≤70)	346	0.771 (0.504–1.176)	0.228
Height (≥170 vs. < 170)	341	0.698 (0.452–1.076)	0.105
BMI (>25 vs. ≤25)	337	0.777 (0.506–1.192)	0.249
Residual tumor (*R*1 & *R*2 vs. *R*0)	345	5.548 (1.789–24.281)	**0.008**
Histologic grade (*G*3 & *G*4 vs. *G*1 & *G*2)	369	2.446 (1.587–3.802)	**<0.001**
Adjacent hepatic tissue inflammation (mild and severe vs. none)	237	1.953 (1.167–3.293)	**0.011**
AFP (ng/ml) (>400 vs. ≤400)	280	3.237 (1.793–6.060)	**<0.001**
Albumin (g/dl) (≥3.5 vs. <3.5)	300	1.045 (0.610–1.794)	0.874
Prothrombin time (>4 vs. ≤4)	297	0.935 (0.568–1.537)	0.790
Child-Pugh grade (*B* & *C* vs. *A*)	241	0.471 (0.174–1.163)	0.115
Fibrosis Ishak score (1/2 & 3/4 & 5/6 vs. 0)	215	1.233 (0.702–2.180)	0.467
Vascular invasion (yes vs. no)	318	1.687 (1.060–2.702)	**0.028**

**Table 4:** Association of OS and clinical characteristics in TCGA HCC patients (Cox regression).

Characteristics	Total(N)	Univariate analysis		Multivariate analysis	
		Hazard ratio (95% CI)	*P* value	Hazard ratio (95% CI)	*P* value
*T* stage (*T*3 & *T*4 & *T*2 vs. *T*1)	370	2.126 (1.481–3.052)	**<0.001**	1.261 (0.765–2.078)	0.363
Pathologic stage (Stage III & Stage IV vs. Stage I & Stage II)	349	2.504 (1.727–3.631)	**<0.001**	1.866 (1.142–3.047)	**0.013**
Tumor status (with tumor vs. tumor free)	354	2.317 (1.590–3.376)	**<0.001**	1.783 (1.193–2.666)	**0.005**
Gender (male vs. female)	373	0.793 (0.557–1.130)	0.200		
Race (Asian and black or African American vs. white)	361	0.791 (0.551–1.135)	0.203		
Age (>60 vs. ≤60)	373	1.205 (0.850–1.708)	0.295		
Weight (>70 vs. ≤70)	345	0.941 (0.657–1.346)	0.738		
Height (≥170 vs. <170)	340	1.232 (0.849–1.788)	0.272		
BMI (>25 vs. ≤25)	336	0.798 (0.550–1.158)	0.235		
Residual tumor (*R*1 & *R*2 vs. *R*0)	344	1.604 (0.812–3.169)	0.174		
Histologic grade (*G*3 & *G*4 vs. *G*1 & *G*2)	368	1.091 (0.761–1.564)	0.636		
Adjacent hepatic tissue inflammation (mild and severe vs. none)	236	1.194 (0.734–1.942)	0.475		
AFP (ng/ml) (>400 vs. ≤400)	279	1.075 (0.658–1.759)	0.772		
Albumin(g/dl) (≥3.5 vs. <3.5)	299	0.897 (0.549–1.464)	0.662		
Prothrombin time (>4 vs. ≤4)	296	1.335 (0.881–2.023)	0.174		
Child-Pugh grade (*B* & *C* vs. *A*)	240	1.643 (0.811–3.330)	0.168		
Fibrosis Ishak score (1/2 & 3/4 & 5/6 vs. 0)	214	0.772 (0.465–1.281)	0.316		
Vascular invasion (yes vs. no)	317	1.344 (0.887–2.035)	0.163		
ELF3-AS1 (high vs. low)	373	1.841 (1.292–2.624)	**<0.001**	1.667 (1.127–2.468)	**0.011**

**Table 5:** Gene sets enriched in of ELF3-AS1 high and low expression groups in HCC (GSEA).

Description	NES	*P* adjust	FDR
REACTOME_GPCR_LIGAND_BINDING	1.508	0.020	0.017
REACTOME_M_PHASE	1.594	0.020	0.017
REACTOME_CLASS_A_1_RHODOPSIN_LIKE_RECEPTORS_	1.456	0.020	0.017
REACTOME_CELL_CYCLE_CHECKPOINTS	1.703	0.020	0.017
REACTOME_TRANSLATION	1.804	0.020	0.017
REACTOME_MITOTIC_METAPHASE_AND_ANAPHASE	1.784	0.020	0.017
REACTOME_SIGNALING_BY_ROBO_RECEPTORS	1.810	0.020	0.017
REACTOME_KERATINIZATION	1.750	0.020	0.017
REACTOME_RRNA_PROCESSING	1.836	0.020	0.017

**Table 6:** Correlation between ELF3-AS1 expression in HCC and immune cells.

LncRNA name	Cell type	Correlation coefficient (Spearman)	*P* value (Spearman)
ELF3-AS1	aDC	0.158	0.002^*∗*^
ELF3-AS1	B cells	0.151	0.004^*∗*^
ELF3-AS1	CD8 T cells	−0.169	0.001^*∗*^
ELF3-AS1	Cytotoxic cells	−0.104	0.045^*∗*^
ELF3-AS1	DC	−0.055	0.29
ELF3-AS1	Eosinophils	−0.161	0.002^*∗*^
ELF3-AS1	iDC	0.079	0.127
ELF3-AS1	Macrophages	0.182	<0.001^*∗*^
ELF3-AS1	Mast cells	−0.046	0.378
ELF3-AS1	Neutrophils	−0.181	<0.001^*∗*^
ELF3-AS1	NK CD56bright cells	0.29	<0.001^*∗*^
ELF3-AS1	NK CD56dim cells	−0.093	0.072
ELF3-AS1	NK cells	−0.165	0.001^*∗*^
ELF3-AS1	pDC	0.018	0.722
ELF3-AS1	T cells	0.105	0.042^*∗*^
ELF3-AS1	T helper cells	−0.006	0.912
ELF3-AS1	Tcm	−0.27	<0.001^*∗*^
ELF3-AS1	Tem	0.013	0.808
ELF3-AS1	TFH	0.245	<0.001^*∗*^
ELF3-AS1	Tgd	−0.101	0.05
ELF3-AS1	Th1 cells	0.095	0.065
ELF3-AS1	Th17 cells	−0.152	0.003^*∗*^
ELF3-AS1	Th2 cells	0.383	<0.001^*∗*^
ELF3-AS1	TReg	−0.124	0.017^*∗*^

## Data Availability

The data used to support the findings of this study are available from the corresponding author upon request.

## References

[1] Siegel R. L., Miller K. D., Jemal A. (2020). Cancer statistics, 2020. *CA: A Cancer Journal for Clinicians*.

[2] Gupta M., Gabriel H., Miller F. H. (2018). Role of imaging in surveillance and diagnosis of hepatocellular carcinoma. *Gastroenterology Clinics of North America*.

[3] Dufour J.-F., Johnson P. (2010). Liver cancer: from molecular pathogenesis to new therapies. *Journal of Hepatology*.

[4] Du J., Chen M., Liu J., Hu P., Guan H., Jiao X. (2019). LncRNA F11‐AS1 suppresses liver hepatocellular carcinoma progression by competitively binding with miR‐3146 to regulate PTEN expression. *Journal of Cellular Biochemistry*.

[5] Grandhi M. S., Kim A. K., Ronnekleiv-Kelly S. M., Kamel I. R., Ghasebeh M. A., Pawlik T. M. (2016). Hepatocellular carcinoma: from diagnosis to treatment. *Surgical Oncology*.

[6] Jiao Y., Li Y., Ji B., Cai H., Liu Y. (2019). Clinical value of lncRNA LUCAT1 expression in liver cancer and its potential pathways. *Journal of Gastrointestinal and Liver Diseases*.

[7] Ricciuti B., Mencaroni C., Paglialunga L. (2016). Long noncoding RNAs: new insights into non-small cell lung cancer biology, diagnosis and therapy. *Medical Oncology*.

[8] Huarte M. (2015). The emerging role of lncRNAs in cancer. *Nature Medicine*.

[9] Zhang Z., Wang S., Liu Y., Meng Z., Chen F. (2019). Low lncRNA ZNF385D-AS2 expression and its prognostic significance in liver cancer. *Oncology Reports*.

[10] Xiao S., Huang S., Yang J. (2020). Overexpression of GIHCG is associated with a poor prognosis and immune infiltration in hepatocellular carcinoma. *OncoTargets and Therapy*.

[11] Guo Y., Chen D., Su X., Chen J., Li Y. (2019). The lncRNA ELF3-AS1 promotes bladder cancer progression by interaction with Krüppel-like factor 8. *Biochemical and Biophysical Research Communications*.

[12] Chu H., Li Z., Gan Z., Yang Z., Wu Z., Rong M. (2019). LncRNA ELF3-AS1 is involved in the regulation of oral squamous cell carcinoma cell proliferation by reprogramming glucose metabolism. *OncoTargets and Therapy*.

[13] Yuan J., Kang J., Yang M. (2020). Long non-coding RNA ELF3-antisense RNA 1 promotes osteosarcoma cell proliferation by upregulating Kruppel-like factor 12 potentially via methylation of the microRNA-205 gene. *Oncology Letters*.

[14] Zhang Z., Nong L., Chen M. L. (2020). LncRNA ELF3-AS1 promotes nonsmall cell lung cancer cell invasion and migration by downregulating miR-212. *Cancer Biother Radiopharm*.

[15] Mei J. C., Yan G., Mei S. Q. (2020). Diagnostic and prognostic potentials of long noncoding RNA ELF3-AS1 in glioma patients. *Disease Markers*.

[16] Liu J., Lichtenberg T., Hoadley K. A. (2018). An integrated TCGA pan-cancer clinical data resource to drive high-quality survival outcome analytics. *Cell*.

[17] Love M. I., Huber W., Anders S. (2014). Moderated estimation of fold change and dispersion for RNA-seq data with DESeq2. *Genome Biology*.

[18] Yu G., Wang L.-G., Han Y., He Q.-Y. (2012). clusterProfiler: an R package for comparing biological themes among gene clusters. *OMICS: A Journal of Integrative Biology*.

[19] Subramanian A., Tamayo P., Mootha V. K. (2005). Gene set enrichment analysis: a knowledge-based approach for interpreting genome-wide expression profiles. *Proceedings of the National Academy of Sciences*.

[20] Hänzelmann S., Castelo R., Guinney J. (2013). GSVA: gene set variation analysis for microarray and RNA-seq data. *BMC Bioinformatics*.

[21] Bindea G., Mlecnik B., Tosolini M. (2013). Spatiotemporal dynamics of intratumoral immune cells reveal the immune landscape in human cancer. *Immunity*.

[22] Zhou T., Lin K., Nie J. (2021). LncRNA SPINT1-AS1 promotes breast cancer proliferation and metastasis by sponging let-7 a/b/i-5p. *Pathology - Research and Practice*.

[23] Granito A., Muratori L., Lalanne C. (2021). Hepatocellular carcinoma in viral and autoimmune liver diseases: role of CD4+ CD25+ Foxp3+ regulatory T cells in the immune microenvironment. *World Journal of Gastroenterology*.

